# Histopathological and Molecular Insights into Chronic Nasopharyngeal and Otic Disorders in Children: Structural and Immune Mechanisms Underlying Disease Chronicity

**DOI:** 10.3390/life15081228

**Published:** 2025-08-03

**Authors:** Diana Szekely, Flavia Zara, Raul Patrascu, Cristina Stefania Dumitru, Dorin Novacescu, Alexia Manole, Carmen Aurelia Mogoanta, Dan Iovanescu, Gheorghe Iovanescu

**Affiliations:** 1 Doctoral School, Victor Babes University of Medicine and Pharmacy Timisoara, E. Murgu Square, No. 2, 300041 Timisoara, Romania; diana.szekely@umft.ro; 2 Department II of Microscopic Morphology, Discipline of Histology, “Victor Babes” University of Medicine and Pharmacy Timisoara, E. Murgu Square, No. 2, 300041 Timisoara, Romania; flavia.zara@umft.ro (F.Z.); novacescu.dorin@umft.ro (D.N.); 3 Department of Functional Sciences, ‘Victor Babes’ University of Medicine and Pharmacy, 300041 Timisoara, Romania; patrascu.raul@umft.ro; 4 Faculty of Medicine and Pharmacy, University of Oradea, 410087 Oradea, Romania; manole.alexia@student.uoradea.ro; 5 Department of Otorhinolaryngology, University of Medicine and Pharmacy of Craiova, 200349 Craiova, Romania; carmen.mogoanta@umfcv.ro; 6 ENT Department, “Victor Babes” University of Medicine and Pharmacy Timisoara, Eftimie Murgu Square No. 2, 300041 Timisoara, Romania; dan.iovanescu@umft.ro (D.I.); giovanescu@umft.ro (G.I.)

**Keywords:** chronic nasopharyngeal disorders, chronic otic disorders, histopathology, molecular mechanisms, pediatric ENT, immune dysregulation, epithelial remodeling, tissue fibrosis, mucosal barrier, inflammation

## Abstract

Chronic nasopharyngeal and otic disorders in children represent a significant clinical challenge due to their multifactorial etiology, variable presentation, and frequent resistance to standard therapies. Although often approached from a symptomatic or anatomical perspective, these conditions are deeply rooted in histological and molecular alterations that sustain inflammation, impair mucosal function, and promote recurrence. This narrative review synthesizes the current knowledge on the normal histology of the nasopharynx, Eustachian tube, and middle ear, and explores key pathophysiological mechanisms, including epithelial remodeling, immune cell infiltration, cytokine imbalance, and tissue fibrosis. Special emphasis is placed on the role of immunohistochemistry in defining inflammatory phenotypes, barrier dysfunction, and remodeling pathways. The presence of biofilm, epithelial plasticity, and dysregulated cytokine signaling are also discussed as contributors to disease chronicity. These findings have direct implications for diagnosis, therapeutic stratification, and postoperative monitoring. By integrating histological, immunological, and molecular data, clinicians can better characterize disease subtypes, anticipate treatment outcomes, and move toward a more personalized and biologically informed model of pediatric ENT care.

## 1. Introduction

Chronic nasopharyngeal and otic disorders are among the most prevalent conditions affecting children worldwide, often presenting with nonspecific symptoms, such as nasal obstruction, recurrent otitis media, mouth breathing, and sleep disturbances. While these disorders are frequently approached from a clinical or anatomical perspective, emerging evidence underscores the central role of histological, immunological, and molecular alterations in their pathogenesis, persistence, and recurrence [1,2].

The nasopharynx and middle ear are contiguous structures lined by specialized respiratory epithelium, rich in immune-active cells and mucosa-associated lymphoid tissue (MALT). These tissues form a dynamic barrier against inhaled pathogens and allergens, mediating immune surveillance and maintaining mucociliary clearance. In chronic disease states, however, this finely regulated system becomes disrupted. Histopathological findings frequently reveal epithelial metaplasia, lymphoid follicular hyperplasia, goblet cell proliferation, and subepithelial fibrosis, reflecting chronic inflammation and mucosal remodeling [3,4,5]. In the middle ear, similar changes accompany Eustachian tube dysfunction and contribute to effusion persistence and conductive hearing loss [6,7].

At the molecular level, pro-inflammatory cytokines, such as interleukin (IL)-1β, IL-6, tumor necrosis factor alpha (TNF-α), and transforming growth factor beta (TGF-β) drive immune activation, tissue remodeling, and epithelial barrier dysfunction [8]. Pattern recognition receptors (e.g., TLR2, TLR4) expressed on epithelial and immune cells detect microbial components and perpetuate local inflammation. Furthermore, matrix metalloproteinases (MMPs), particularly MMP-2 and MMP-9, are overexpressed in chronically inflamed adenoids and tympanic mucosa, contributing to extracellular matrix degradation and tissue disorganization [9,10,11].

In children, these processes are potentiated by anatomical and immunological immaturity, environmental exposures (such as tobacco smoke and air pollution), and recurrent infections. Studies have also emphasized the role of microbial biofilms in sustaining low-grade inflammation and resistance to treatment in both adenoidal tissue and the middle ear [12,13].

Despite the increasing volume of data on these mechanisms, a significant knowledge gap persists among clinicians, many of whom rely solely on symptomatology and radiographic findings when managing pediatric upper airway pathology. This review aims to bridge that gap by offering a comprehensive synthesis of the histopathological and molecular foundations of chronic nasopharyngeal and otic diseases. Understanding these mechanisms is not a purely academic endeavor—it directly informs therapeutic decision-making, helps identify refractory cases, supports patient stratification, and opens avenues for personalized, immune-targeted interventions [14,15,16].

Incorporating structural and immunopathological knowledge into clinical reasoning enhances diagnostic precision and guides the selection of appropriate medical or surgical strategies. Ultimately, an integrated understanding of tissue-level changes empowers otolaryngologists, pediatricians, and family physicians to deliver more effective, individualized, and durable care for affected children.

This review was conducted as a narrative synthesis of current evidence and does not follow a systematic review protocol, such as PRISMA. References were selected based on relevance, recency, and their contribution to understanding the histopathological and immunological mechanisms underlying chronic pediatric ENT disorders.

## 2. Normal Histology of the Nasopharynx, Eustachian Tube, and Middle Ear

An accurate understanding of the normal histological architecture of the nasopharynx, Eustachian tube, and middle ear is essential to recognizing the cellular and tissue-level alterations that characterize chronic pediatric pathology. These anatomical compartments are structurally continuous and functionally integrated, forming a mucosal interface exposed to a high microbial load and environmental stimuli. The specialized epithelium, subepithelial immune structures, glandular systems, and vascular networks collaborate to maintain homeostasis and protect against infection and inflammation [2].

### 2.1. Nasopharyngeal Epithelium and Mucosal Immunity

The nasopharynx is lined by pseudostratified ciliated columnar epithelium rich in goblet cells, which produce mucins that maintain hydration and entrap inhaled particles. Beneath the epithelial layer lies the lamina propria, populated with lymphocytes, plasma cells, and antigen-presenting cells (APCs), embedded in a loose connective tissue stroma [17]. Numerous seromucous glands within the lamina propria secrete antimicrobial compounds including lysozyme, lactoferrin, and secretory IgA [18].

**Figure 1:**
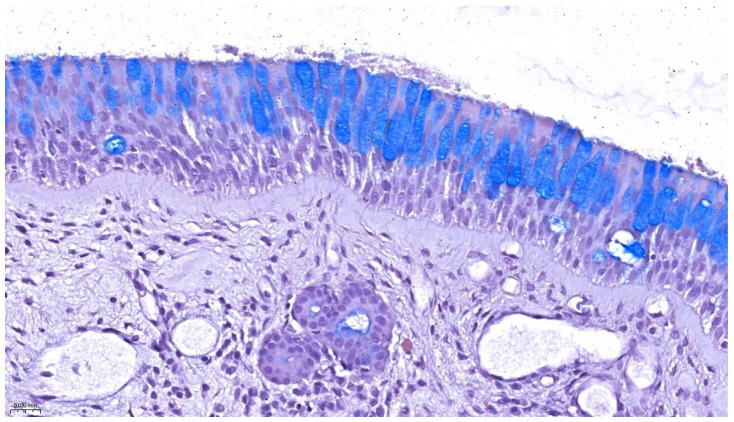
Normal respiratory-type epithelium of the inferior nasal turbinate. Alcian Blue–PAS staining highlights goblet cells (blue) within a pseudostratified ciliated columnar epithelium. The underlying lamina propria shows a well-vascularized loose connective tissue with mucous glands and scattered immune cells. Original magnification: 40×.

Histological examination of the normal respiratory mucosa reveals a highly specialized pseudostratified ciliated columnar epithelium, integral to mucociliary clearance and host defense. Goblet cells, interspersed among ciliated epithelial cells, are responsible for mucin production, ensuring continuous hydration and trapping of inhaled particles. The underlying lamina propria contains loose connective tissue, numerous blood vessels, and seromucous glands, which supplement mucosal hydration and antimicrobial activity. This organization is clearly illustrated in Figure 1, which shows a well-preserved segment of nasal respiratory epithelium stained with Alcian Blue-PAS (periodic acid–Schiff), highlighting the mucopolysaccharide content of goblet cell secretions and the structural integrity of the epithelial barrier.

A key histological component of the nasopharynx is the mucosa-associated lymphoid tissue (MALT), most prominently represented by the pharyngeal tonsil (adenoids), part of Waldeyer’s ring. The adenoidal tissue contains organized lymphoid follicles with germinal centers surrounded by interfollicular T-cell zones and is overlaid by a specialized follicle-associated epithelium (FAE). This FAE contains microfold (M) cells, which lack cilia and goblet cells but excel at transcytosis of antigens from the lumen to the underlying immune cells [3,19].

This immune architecture supports a finely balanced state of immune vigilance and tolerance, allowing response to pathogens while limiting overactivation. In children, the adenoids are particularly active due to early life antigenic exposure, but their hypertrophy can impair nasal airflow and contribute to Eustachian tube dysfunction [5].

### 2.2. Eustachian Tube: Segmental Architecture and Functional Morphology

The Eustachian tube (ET) connects the middle ear to the nasopharynx and consists of two anatomically distinct segments, namely the medial cartilaginous portion and the lateral bony portion. The cartilaginous segment, constituting the proximal two-thirds, is lined by respiratory-type pseudostratified ciliated columnar epithelium with abundant goblet cells and underlying seromucous glands. This portion is the main site of mucociliary clearance and immunologic interaction with nasopharyngeal stimuli [20].

The bony segment, which opens into the tympanic cavity, shows a gradual transition to cuboidal or squamous epithelium with reduced secretory and ciliary capacity. The subepithelial stroma in this portion contains fewer lymphoid aggregates and less glandular tissue, reflecting its more passive role in middle ear physiology [6].

Ciliary action within the ET propels mucus toward the nasopharynx, clearing potential pathogens and preventing retrograde contamination. ET opening during swallowing or yawning is coordinated by the tensor veli palatini and levator veli palatini muscles. In infants and young children, the ET is shorter, more horizontally oriented, and more compliant, which impairs clearance and promotes pathogen migration—a key anatomical risk factor for otitis media [21].

### 2.3. Middle Ear Mucosa and Cellular Composition

The middle ear cavity is lined by a thin mucosa composed of simple cuboidal to low columnar epithelium. In the mesotympanum, this lining is typically smooth and paucicellular, while the epitympanum and hypotympanum may display focal squamous metaplasia, especially in response to chronic irritation [22]. Under physiological conditions, the middle ear mucosa has a limited immune role, but it possesses basic defense mechanisms including tight junctions, mucus secretion, and localized lymphoid cell populations.

In the pediatric population, the middle ear mucosa is often thicker and more vascularized, with a greater number of secretory cells than in adults. These histological features correlate with the higher incidence of effusions and infections in children [11].

In the absence of inflammation, goblet cells and ciliated epithelium are sparse. However, chronic stimulation, as seen in Eustachian tube dysfunction or biofilm exposure, leads to epithelial hyperplasia, increased mucus production, and recruitment of immune cells to the submucosa. Understanding these baseline features is essential for interpreting pathological changes observed in chronic otitis media [23].

## 3. Histopathological Features in Chronic Disease

Chronic inflammation of the nasopharynx and middle ear triggers a cascade of cellular and tissue-level changes that progressively alter the normal histoarchitecture. These histopathological modifications not only sustain symptomatology but also contribute to therapeutic resistance and disease recurrence. Recognizing specific patterns of remodeling, immune infiltration, and epithelial adaptation is crucial for distinguishing active versus quiescent pathology, identifying surgical indications, and informing immunomodulatory therapies.

### 3.1. Adenoidal Hypertrophy and Nasopharyngeal Inflammation

Adenoidal hypertrophy is one of the most frequent histopathological findings in pediatric patients with upper airway obstruction or Eustachian tube dysfunction. Microscopically, the adenoids display enlarged lymphoid follicles with prominent germinal centers surrounded by interfollicular zones rich in T lymphocytes and plasma cells. The follicle-associated epithelium (FAE), responsible for antigen sampling, often undergoes squamous metaplasia and displays focal areas of epithelial erosion in chronic inflammatory states [3].

**Figure 2:**
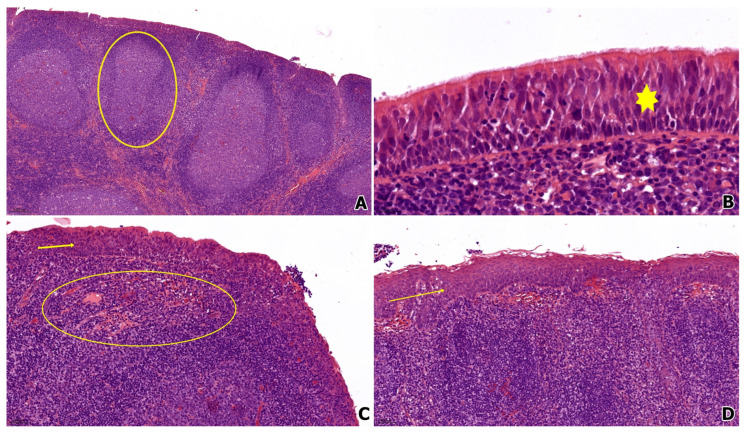
Histopathological progression in chronic adenoiditis. (**A**) Low-power view (10×) showing adenoidal lymphoid hyperplasia with prominent germinal centers (circled); (**B**) ciliated pseudostratified respiratory epithelium (star) (40×); (**C**) transitional zone displaying epithelial metaplasia (arrow) and dense subepithelial chronic inflammatory infiltrate (circled) (20×); (**D**) surface lining replaced by non-keratinized stratified squamous epithelium (arrow), consistent with advanced epithelial remodeling (20×). Hematoxylin and eosin staining.

The histopathological continuum of chronic adenoiditis is characterized by both lymphoid and epithelial remodeling. As illustrated in Figure 2, adenoidal hypertrophy is marked by prominent lymphoid follicles with active germinal centers (panel A), consistent with chronic antigenic stimulation. The overlying epithelium varies significantly depending on disease chronicity. In panel B, the respiratory epithelium retains its ciliated pseudostratified morphology (highlighted by the star), representing early or partially preserved mucosal architecture. However, in panel C, areas of epithelial metaplasia are evident (arrow), showing loss of cilia and progressive transformation toward a stratified pattern. This transition is accompanied by a dense subepithelial chronic inflammatory infiltrate (circled), composed predominantly of lymphocytes and plasma cells. In more advanced lesions (panel D), the surface epithelium exhibits a complete conversion to non-keratinized stratified squamous epithelium (arrow), lacking goblet cells or cilia, indicative of long-standing irritation and epithelial adaptation. This metaplastic transformation compromises mucociliary function and may perpetuate antigenic exposure and chronicity.

The subepithelial stroma is infiltrated with polymorphonuclear leukocytes, macrophages (CD68+), and dendritic cells, along with hypertrophic seromucous glands and increased vascularization. In cases of allergic rhinitis or eosinophilic inflammation, a marked infiltration of eosinophils and mast cells (tryptase+) can be observed, often accompanied by increased deposition of extracellular matrix proteins [4].

Chronic antigenic stimulation and biofilm presence in adenoidal crypts further perpetuate inflammation, as demonstrated by scanning electron microscopy studies that reveal extensive bacterial colonies encased in extracellular polymeric substances (EPS), contributing to immunological exhaustion and reduced response to antibiotics [24].

Recent evidence also highlights the role of resident mesenchymal stem cells (MSCs) in mucosal remodeling and tissue repair in chronic nasal disease. MSCs isolated from healthy nasal mucosa exhibit multilineage differentiation potential—into adipocytes, chondrocytes, and osteoblasts—and express characteristic markers, such as CD73, CD90, and CD105, while lacking hematopoietic lineage markers (CD14, CD34, and CD45). In contrast, MSCs derived from pathological tissues, including Killian’s polyps and nasal polyps associated with chronic rhinosinusitis, demonstrate impaired adipogenic and osteogenic differentiation capacity, increased apoptosis, and reduced regenerative function. This dysfunction may contribute to persistent epithelial damage and impaired tissue repair in chronic inflammatory settings [25,26].

### 3.2. Middle Ear Mucosal Remodeling in Otitis Media

The middle ear mucosa undergoes profound structural remodeling in chronic otitis media (COM), particularly in otitis media with effusion (OME) and adhesive otitis. The epithelial lining transforms from a cuboidal monolayer into a thicker, pseudostratified epithelium with abundant goblet cells and variable degrees of squamous metaplasia. Ciliated cell density decreases significantly, while mucus-producing cells dominate the epithelium, impairing normal mucociliary clearance [27].

The subepithelial layer becomes fibrotic, with increased deposition of collagen types I and III, neovascularization, and infiltration of chronic inflammatory cells. Macrophages, plasma cells, and neutrophils are common, particularly in regions of active inflammation. Overexpression of MMP-9 and TGF-β is frequently documented in these lesions, contributing to extracellular matrix degradation and tissue remodeling [28].

In cases of chronic suppurative otitis media (CSOM), cholesteatoma formation may occur, characterized by keratinizing stratified squamous epithelium that invades the middle ear cleft, accompanied by perimatrix inflammation, granulation tissue, and bone resorption. The molecular environment is dominated by elevated IL-1β, TNF-α, and oxidative stress markers that promote osteoclast activation and ossicular erosion [23].

### 3.3. Epithelial Barrier Dysfunction and Metaplastic Changes

Chronic inflammation compromises the epithelial barrier integrity of both nasopharyngeal and middle ear mucosa. Tight junction proteins, such as occludin, claudin-1, and ZO-1, are downregulated in chronic disease states, leading to increased paracellular permeability and susceptibility to pathogen invasion [29]. Concurrently, epithelial cells exhibit altered differentiation patterns, transitioning from a ciliated phenotype to mucus-secreting or squamous phenotypes under inflammatory stress, a process regulated by Notch, TGF-β, and IL-13 signaling pathways [30].

Histological studies have also demonstrated a reduction in epithelial proliferation markers, such as FOXJ1 (required for cilia formation) and Ki-67, in severely damaged mucosa, indicating impaired regenerative capacity and chronic epithelial fatigue [31,32].

### 3.4. Immune Cell Infiltrates and Chronicity Markers

Quantitative immunohistochemical (IHC) analyses show that chronic ENT lesions are characterized by increased densities of CD3+ T cells, CD20+ B cells, and CD138+ plasma cells. These populations are organized in follicular or diffuse patterns, depending on the antigenic load and chronicity of inflammation. In eosinophilic variants (e.g., allergic rhinitis and eosinophilic CRS), IL-5 and IL-13 expressions are elevated, while neutrophilic inflammation (e.g., biofilm-related disease) correlates with IL-8 and MMP-9 overexpression [33,34].

Persistent expression of pro-inflammatory cytokines sustains a loop of immune cell recruitment, tissue damage, and delayed resolution. In addition, the local microenvironment becomes increasingly hypoxic due to impaired ventilation and vascular congestion. Hypoxia-inducible factors (HIF-1α) promote angiogenesis and fibrotic responses, further entrenching the chronic disease state [35].

These histopathological changes collectively reflect a progressive loss of epithelial and mucosal homeostasis, ultimately altering both function and therapeutic response. A comparative overview of the most relevant histological differences between normal and chronically inflamed nasopharyngeal and middle ear mucosa is presented in Table 1.

## 4. Molecular Mechanisms of Inflammation and Remodeling

The transition from acute to chronic nasopharyngeal and otic inflammation is sustained by a complex molecular network involving epithelial–immune interactions, pro-inflammatory signaling pathways, and aberrant tissue repair responses. While histological features reflect visible manifestations of chronicity, the underlying molecular mechanisms provide essential insight into disease persistence, refractoriness to therapy, and potential targets for personalized intervention.

### 4.1. Cytokine Profiles and Inflammatory Signaling Pathways

In chronic ENT pathology, cytokine expression is persistently dysregulated. The pro-inflammatory milieu is dominated by interleukin (IL)-1β, IL-6, and tumor necrosis factor-alpha (TNF-α), which mediate leukocyte recruitment, epithelial activation, and upregulation of adhesion molecules. These cytokines enhance vascular permeability and stimulate fibroblast proliferation, leading to stromal expansion and remodeling [8,36].

Additionally, chemokines, such as IL-8 and monocyte chemoattractant protein-1 (MCP-1), drive the accumulation of neutrophils and monocytes, particularly in neutrophil-dominant forms of chronic otitis media or adenoiditis. Their continuous expression fosters tissue damage and impairs resolution, especially in the presence of microbial persistence or biofilms [33,37].

A shift toward Th2 polarization—characterized by elevated IL-4, IL-5, and IL-13—is common in allergic or eosinophilic phenotypes. These cytokines promote eosinophil survival, IgE production, and goblet cell hyperplasia, particularly within adenoidal and nasal tissues. In contrast, Th17-associated cytokines (e.g., IL-17A and IL-22) have been implicated in neutrophilic inflammation and epithelial barrier disruption, particularly in non-atopic children [29,38].

### 4.2. Pattern Recognition Receptors and Epithelial Activation

The mucosal epithelium acts as an immunologically active barrier by recognizing microbial products through pattern recognition receptors (PRRs), particularly Toll-like receptors (TLRs). TLR2 and TLR4, expressed on epithelial and immune cells, detect peptidoglycan and lipopolysaccharide, respectively, initiating NF-κB-dependent transcription of inflammatory mediators [39].

In chronic ENT disorders, persistent stimulation of TLRs by biofilm-derived ligands leads to continuous activation of the innate immune system and failure to restore mucosal quiescence. This phenomenon is particularly well-documented in the adenoids of children with chronic rhinosinusitis or OME, where enhanced TLR4 expression correlates with disease severity and inflammatory cell infiltration [12,40].

Moreover, dysregulated epithelial responses contribute to increased expression of S100 proteins, defensins, and reactive oxygen species (ROS), which further compromise barrier integrity and promote tissue injury [41].

### 4.3. Tissue Remodeling Mediators: MMPs, TGF-β, and Fibrosis-Associated Pathways

Chronic inflammation stimulates matrix remodeling and fibrosis through the overproduction of matrix metalloproteinases (MMPs), most notably MMP-2 and MMP-9. These enzymes degrade collagen and other extracellular matrix (ECM) proteins, facilitating leukocyte migration but also contributing to tissue destruction when unopposed by tissue inhibitors of metalloproteinases (TIMPs) [42].

Transforming growth factor-beta (TGF-β) is a central driver of fibrotic remodeling in both the adenoids and middle ear mucosa. It promotes fibroblast proliferation, myofibroblast differentiation, and ECM deposition, leading to thickening of the lamina propria and submucosal fibrosis. This profibrotic environment is associated with tympanic membrane retraction, adhesion formation, and Eustachian tube dysfunction in chronic otitis media [4,43].

In addition, fibroblast growth factors (FGFs), vascular endothelial growth factor (VEGF), and hypoxia-inducible factor 1-alpha (HIF-1α) orchestrate neoangiogenesis and tissue repair. While initially beneficial, their persistent expression in poorly ventilated, inflamed tissue may lead to pathological angiogenesis and promote chronicity [44].

### 4.4. Barrier Dysfunction and Epithelial Plasticity

Epithelial integrity is compromised in chronic disease by cytokine- and ROS-induced downregulation of tight junction proteins, such as claudin-1, occludin, and ZO-1. This loss of barrier function facilitates translocation of antigens and pathogens into the subepithelial compartment, perpetuating immune activation [17]. Furthermore, epithelial plasticity—driven by Notch, TGF-β, and Wnt/β-catenin signaling—favors a shift from a ciliated to a mucus-secreting or squamous phenotype. These changes reduce mucociliary clearance efficiency and impair regeneration of functional epithelia. In particular, downregulation of FOXJ1, a transcription factor essential for cilia formation, has been documented in metaplastic middle ear epithelium [31].

### 4.5. Epigenetic and Microbiome-Immune Interactions

Emerging data suggests that chronic mucosal inflammation is modulated by epigenetic mechanisms, such as DNA methylation and histone modification. These changes affect the expression of cytokines, TLRs, and junctional proteins, potentially explaining persistent mucosal reactivity despite antigen removal or therapy [45].

The interaction between the host immune system and local microbiota also plays a pivotal role in disease persistence. Dysbiosis—defined by the overgrowth of pathogenic strains, such as Haemophilus influenzae or Moraxella catarrhalis—promotes sustained TLR activation, increased mucin production, and mucosal remodeling. Restoration of microbiota balance through targeted therapies remains an area of active investigation [46].

In addition to the well-established role of eosinophils as a source of IL-5 and IL-13 in chronic nasal inflammation, recent clinical data support the therapeutic utility of monoclonal antibodies targeting these pathways in eosinophilic chronic rhinosinusitis (ECRS) and chronic rhinosinusitis with nasal polyps (CRSwNP). For instance, benralizumab (anti-IL-5Rα) has shown significant reductions in nasal polyp score (NPS), nasal obstruction severity, olfactory dysfunction, and SNOT-22 scores, including in patients with comorbid severe eosinophilic asthma [47]. Omalizumab (anti-IgE) has demonstrated efficacy in phase III trials (POLYP 1/POLYP 2) and real-world settings for reducing polyp size and improving sinonasal symptoms [48], while dupilumab (anti-IL-4Rα, blocking IL-4/IL-13) has produced sustained improvements in symptom scores, olfaction, and quality of life in CRSwNP patients [49]. Although data on pediatric use are limited, early phase trials, such as the pediatric TATE study, support the pharmacokinetics, safety, and eosinophil-depleting efficacy of benralizumab in children aged 6–11 with severe asthma [50,51]. Moreover, omalizumab and dupilumab are already approved and used in certain pediatric populations with severe allergic asthma and nasal polyposis, with emerging real-world evidence of benefit in CRS phenotypes [52]. These findings suggest that biologic agents targeting IL-5, IgE, and IL-4/IL-13 pathways represent promising adjuncts to standard care in both adult and pediatric eosinophilic nasal disease.

## 5. Immunohistochemistry and Molecular Markers

Immunohistochemistry (IHC) has become an indispensable tool in the evaluation of chronic nasopharyngeal and otic disorders, allowing for the precise characterization of inflammatory profiles, cellular composition, and structural remodeling processes. IHC complements routine histology by revealing subtle immunological and epithelial changes that may otherwise go undetected, especially in complex or treatment-resistant cases [53].

In the context of chronic inflammation, the immune infiltrate often displays a characteristic pattern. T lymphocytes, identified by CD3 staining, are typically found in interfollicular regions of the adenoids and diffusely distributed within inflamed middle ear mucosa. These T-cell populations reflect ongoing cellular immune responses and are frequently associated with chronic antigenic exposure, including viral triggers and bacterial biofilms [54]. B lymphocytes, marked by CD20, are predominantly located in germinal centers, indicative of an active humoral response, while plasma cells, detected through CD138, are particularly abundant in longstanding inflammatory lesions [55]. In cases with allergic or eosinophilic involvement, additional markers, such as major basic protein (MBP) and eosinophil peroxidase (EPO), confirm the presence of eosinophils, whereas mast cells are highlighted using anti-tryptase staining. These patterns are especially relevant in children with underlying allergic diathesis or comorbid asthma [56,57].

Beyond cell identification, immunohistochemistry also enables the profiling of cytokine expression within the inflamed mucosa. Th2-associated cytokines, including interleukin (IL)-5 and IL-13, are frequently elevated in eosinophilic adenoidal or sinonasal inflammation, correlating with tissue eosinophilia, goblet cell metaplasia, and corticosteroid responsiveness [58]. Conversely, Th17-type cytokines, such as IL-17A and IL-22, are more commonly expressed in neutrophil-dominant phenotypes, often seen in biofilm-associated chronic otitis media, and are implicated in epithelial barrier disruption and disease chronicity [59]. Pro-inflammatory mediators, such as TNF-α and IL-1β, are frequently overexpressed in areas of active inflammation [60], particularly in cholesteatomatous disease or hypertrophic adenoids with superimposed bacterial colonization. Their presence is associated with mucosal damage, osteolysis, and subepithelial tissue remodeling [61,62].

Tissue remodeling is further reflected in the expression of extracellular matrix-modifying proteins. Matrix metalloproteinase 9 (MMP-9) is a critical effector molecule involved in extracellular matrix degradation and epithelial–mesenchymal transition [63]. It is widely expressed in inflamed adenoidal tissues and middle ear mucosa and has been linked to tympanic membrane retraction, fibrosis, and poor postoperative outcomes. Transforming growth factor-beta (TGF-β1), a central regulator of fibrosis, drives fibroblast activation and collagen deposition, playing a significant role in the pathogenesis of adhesive otitis and submucosal scarring [64]. Additionally, vascular endothelial growth factor (VEGF) is commonly expressed in hypertrophic adenoids, where it contributes to angiogenesis, mucosal edema, and obstruction-related symptoms [65].

Markers of epithelial differentiation and barrier integrity provide further insight into the functional status of the mucosa. FOXJ1, a transcription factor required for motile cilia formation, is downregulated in chronically inflamed and metaplastic epithelium, correlating with impaired mucociliary clearance [66]. E-cadherin, a key component of adherens junctions, is reduced in areas of epithelial dedifferentiation or squamous metaplasia. Tight junction proteins, such as occludin and claudin-1, are also diminished in chronic disease states, reflecting compromised epithelial barrier function and increased susceptibility to antigen penetration [67].

Clinically, immunohistochemistry is most valuable in selected scenarios. These include cases with atypical clinical evolution, suspected neoplastic transformation, or recurrence after adequate medical or surgical therapy. In such contexts, IHC aids in delineating the inflammatory phenotype—whether eosinophilic, neutrophilic, or lymphoplasmacytic—and guides therapeutic adjustments, including the potential use of targeted biologic agents. Furthermore, it enables the evaluation of tissue responsiveness to therapy by tracking changes in cytokine profiles or remodeling markers before and after treatment.

Recent studies employing flow cytometry have significantly improved understanding of the immune cell landscape in both healthy and diseased nasal mucosa. Specific marker panels have enabled detailed profiling of immune populations: T lymphocytes (CD3^+^, CD4^+^, and CD8^+^), regulatory subsets (CD25^+^Foxp3^+^ and CD45RA^−^Foxp3^+^), B cells (CD19^+^), dendritic cells (CD11c^+^), eosinophils (Siglec-8^+^ and CCR3^+^) and monocytes/macrophages (CD14^+^ and CD16^+^) [68,69,70]. For example, flow-assisted analysis of Tregs in CRSwNP revealed a higher proportion of CD3^+^CD4^+^CD25^+^Foxp3^+^ cells in nasal mucosa versus peripheral blood, with an elevated but functionally compromised CD45RA^−^Foxp3^+^ subset associated with disease severity [68]. Other investigations have utilized panels including Siglec-8 and CCR3 to identify eosinophils, demonstrating robust local eosinophilic infiltration in CRSwNP tissue, as well as markers, such as CD11c, CD14, CD16, and CD19, to quantify dendritic cells, monocytes/macrophages and B cells, respectively [68,70]. These studies reinforce the added value of flow cytometry to characterize the nasal mucosal immune microenvironment in allergic rhinitis and CRSwNP, complementing histology and immunohistochemistry approaches.

Furthermore, the increasing clinical relevance of nasal cytology should be acknowledged. As a non-invasive, inexpensive, and easily repeatable technique, it enables in vivo assessment of epithelial and inflammatory cell profiles in the nasal mucosa. Nasal cytology has been increasingly used to classify disease endotypes, monitor therapeutic response, and guide personalized treatment—particularly in pediatric populations [71]. For instance, cellular clinical grading systems that incorporate cytology and comorbidities correlate with recurrence risk of CRSwNP. Mixed cellular phenotypes (eosinophils, mast cells), or neutrophil versus eosinophilic dominance, have prognostic and therapeutic implications. Moreover, cytological assessment has been shown to reflect shifts in inflammatory infiltrate during biologic therapy (e.g., eosinophil decrease under dupilumab) and can, thus, serve as a practical monitoring tool in CRS and related nasal disorders [72].

To support the diagnostic and clinical relevance of immunohistochemical profiling in chronic nasopharyngeal and otic pathology, Table 2 summarizes key markers, their immunologic targets, associated pathological patterns, and potential implications for management. This synthesis illustrates how the immunophenotype of adenoidal and middle ear tissues—ranging from T/B cell density to cytokine signatures and remodeling proteins—can refine diagnostic accuracy, predict therapeutic response, and assist in stratifying patients for medical versus surgical intervention.

While not routinely performed in all cases of chronic ENT disease, immunohistochemistry holds growing promise in both clinical and research settings. Its integration into diagnostic workflows may facilitate earlier recognition of refractory patterns, enhance risk stratification, and support the development of personalized treatment algorithms tailored to the underlying pathophysiology of each patient.

## 6. Diagnostic and Clinical Implications of Histopathologic and Molecular Insights

The integration of histopathological and molecular findings into clinical decision-making is increasingly recognized as a critical component in the management of chronic nasopharyngeal and otic disorders in children. While clinical assessment, endoscopic visualization, and imaging remain essential tools in evaluating these patients, they often fail to capture the underlying biological processes driving disease persistence, therapeutic resistance, or recurrence. The insights gained from tissue-based analyses offer a deeper understanding of pathophysiology and hold significant value for diagnosis, prognosis, and individualized care [4,35].

Histological examination of adenoidal and middle ear tissues can clarify the nature and severity of chronic inflammation. For instance, the predominance of follicular hyperplasia in adenoids supports an antigen-driven response, whereas extensive fibrosis, epithelial metaplasia, or glandular atrophy indicate chronic remodeling and potentially irreversible tissue changes [9,73]. The identification of such features can influence the timing and type of surgical intervention. For example, in children with obstructive sleep apnea secondary to adenoidal hypertrophy, the presence of persistent eosinophilic inflammation or high VEGF expression may predict a suboptimal response to adenoidectomy alone and suggest the need for adjunctive medical management [33,59].

The characterization of inflammatory phenotypes through immunohistochemistry further refines clinical stratification. Eosinophilic adenoiditis or otitis media, defined by IL-5 or IL-13 expression and tissue eosinophilia, are more likely to respond to corticosteroids or anti-IL-5 biologics [74]. Conversely, neutrophil-dominant, biofilm-associated forms of chronic otitis media tend to exhibit poor response to anti-inflammatory agents and may require surgical debridement or long-term topical therapy. Thus, the immunological signature of a lesion can serve as a predictive marker for therapeutic responsiveness and recurrence risk [75].

From a diagnostic perspective, immunohistochemical analysis can be especially helpful in differentiating between infection-, allergy-, and immune-mediated pathologies. For instance, persistent adenoidal enlargement in older children or adolescents warrants careful histological evaluation to exclude lymphoproliferative disorders, nasopharyngeal carcinoma, or granulomatous diseases, such as sarcoidosis or tuberculosis. In these contexts, markers, such as CD3, CD20, Ki-67, or EBER (Epstein–Barr virus-encoded RNA), may provide essential diagnostic clues and direct further oncologic or infectious work-up [75,76].

The recognition of biofilm-associated pathology also has important clinical implications. Histological and electron microscopic evidence of extracellular polymeric matrix structures and sessile bacterial colonies within the adenoids or middle ear mucosa suggest the need for alternative strategies beyond conventional antibiotics. Such cases may benefit from adenoidectomy, topical antimicrobial agents, or even experimental therapies targeting biofilm integrity and epithelial–bacterial interactions. Incorporating these findings into clinical guidelines could improve treatment outcomes, reduce recurrence, and minimize unnecessary antibiotic exposure [9,24].

The molecular pathways underlying chronic inflammation and mucosal remodeling in the nasopharynx and middle ear are multifaceted, involving immune, epithelial, and stromal elements. To provide an integrative perspective, Table 3 summarizes the most relevant pathophysiological mechanisms described in this review, along with their molecular drivers, histologic correlates, and clinical implications. This overview emphasizes how understanding the underlying biology—ranging from barrier dysfunction to cytokine-mediated fibrosis—can inform diagnostic strategies and guide personalized therapeutic approaches in pediatric ENT practice.

In the surgical setting, preoperative or intraoperative histopathological information can guide the extent of resection and the need for adjunct procedures. For instance, in children undergoing tympanoplasty for adhesive otitis media, histological evidence of intense fibrosis and low FOXJ1 expression in the middle ear epithelium may indicate a higher risk of postoperative effusion persistence, prompting closer audiological follow-up and early intervention if required [75,76]. Additionally, reduced expression of FOXJ1, a master regulator of ciliary development, correlates with impaired mucociliary clearance and poorer clinical outcomes in chronic mucosal diseases, suggesting that its low-level detection in middle ear epithelium may predict suboptimal surgical success [77].

Based on the histopathological patterns and immunophenotypes discussed in the previous sections, the identification of specific inflammatory endotypes—such as eosinophilic, neutrophilic, mixed eosinophilic–mast cell, and fibrotic—provides important therapeutic implications. Recognizing these profiles allows clinicians to tailor interventions more precisely, including the selection of anti-inflammatory agents, surgical strategies, or targeted biologic therapies. Table 4 presents a summary of the main inflammatory subtypes encountered in chronic nasopharyngeal and otic disorders in children, along with their histological features, key immunological markers, and recommended therapeutic approaches.

On a broader scale, the incorporation of tissue biomarkers into diagnostic algorithms supports a more nuanced classification of chronic ENT disorders—moving beyond anatomic or symptom-based categories to pathophysiological endotypes. This paradigm shift paves the way for precision medicine in pediatric otolaryngology, where treatment plans are tailored according to the specific molecular and cellular landscape of each patient’s disease.

Importantly, this approach reinforces the role of interdisciplinary collaboration between clinicians, pathologists, and researchers. It encourages the routine submission of surgical specimens for histopathological evaluation—not just as a rule-out step for malignancy, but as a valuable diagnostic and prognostic resource. Moreover, it supports the long-term goal of developing clinically validated biomarkers that can be assessed through less invasive means, such as nasal cytology, mucosal brushings, or fluid analysis.

While histopathological and immunohistochemical analyses of nasal mucosal tissue are recognized for their value in defining CRSwNP endotypes and guiding targeted therapies, their routine implementation in non-malignant ENT practice remains limited. In particular, Alobid et al. proposed a pragmatic checklist for tissue histopathology in CRSwNP to standardize practice across centers, acknowledging that histological evaluations beyond exclusion of malignancy are not yet commonplace [4]. Accordingly, the availability of IHC and molecular marker profiling depends heavily on institutional infrastructure and local practice patterns, which may explain the lack of experience reported by some clinicians in routine ENT settings.

In summary, the clinical implications of histopathological and molecular data extend far beyond academic understanding. They have the potential to transform the diagnostic process, guide evidence-based therapeutic decisions, reduce recurrence rates, and improve long-term outcomes in children with chronic nasopharyngeal and otic disorders. Recognizing these insights as fundamental rather than auxiliary elements of ENT care is essential in advancing toward a more personalized and biologically informed model of practice.

## 7. Conclusions

Chronic nasopharyngeal and otic disorders in children are underpinned by complex histopathological and molecular mechanisms that extend far beyond structural obstruction or infection. Persistent inflammation, epithelial remodeling, immune dysregulation, and biofilm formation drive chronic disease and therapeutic resistance. Histological and immunohistochemical evaluation reveals distinct inflammatory phenotypes, barrier dysfunction, and fibrosis, offering diagnostic clarity and prognostic value. These insights are increasingly essential for guiding individualized management strategies, whether medical, surgical, or combined. Incorporating tissue-level analysis into clinical algorithms enables more accurate stratification, identification of refractory cases, and selection of targeted therapies. Ultimately, a biologically informed approach enhances therapeutic precision and long-term outcomes, reinforcing the need for closer integration of clinical, histological, and molecular data in pediatric ENT care.

## Figures and Tables

**Table 1:** Comparative histological features: normal vs. chronic conditions.

Feature	Normal Nasopharynx/Middle Ear	Chronic Inflammatory State
Epithelial type [22,27]	Pseudostratified ciliated columnar	Squamous/metaplastic or hyperplastic
Goblet cells [2,11]	Sparse, functionally balanced	Hyperplasia, excessive mucus production
Subepithelial stroma [28,33]	Loose connective tissue	Fibrosis, neovascularization, glandular hypertrophy
Immune infiltrate [4,33]	Scattered lymphocytes	Dense T/B cells, plasma cells, eosinophils
Barrier integrity (tight junctions) [29,30]	Preserved	Compromised: reduced claudin/occludin expression

**Table 2:** Key immunohistochemical markers and their interpretation. Abbreviations: IL—interleukin; MMP—matrix metalloproteinase; TGF—transforming growth factor; VEGF—vascular endothelial growth factor; Th—T helper cell subtype.

Marker	Cell Type/Target	Associated Pathology	Clinical Implication
CD3 [55]	T lymphocytes	Chronic cellular immune response	Intensity reflects Th1/Th17 activity
CD20 [56]	B lymphocytes	Follicular hyperplasia in adenoids	Active humoral response
CD138 [56]	Plasma cells	Longstanding inflammation	Supports chronicity
IL-5, IL-13 [58]	Th2 cytokines	Eosinophilic inflammation	Predicts steroid/biologic responsiveness
IL-17A [59]	Th17 cytokine	Neutrophilic, biofilm-associated pathology	Marker of recalcitrant disease
MMP-9 [63]	Matrix degradation enzyme	Tissue remodeling and tympanic retraction	Linked to recurrence
TGF-β [64]	Fibrosis and repair	Adhesive otitis, subepithelial scarring	Indicator of irreversible remodeling
VEGF [65]	Angiogenesis	Adenoidal hypertrophy, mucosal edema	May predict surgical outcome
FOXJ1 [65]	Ciliogenesis marker	Loss of cilia in metaplastic epithelium	Indicates impaired mucociliary clearance
E-cadherin [66]	Adherens junctions	Epithelial dedifferentiation	Barrier integrity assessment
Occludin, Claudin-1 [29]	Tight junction proteins	Epithelial barrier dysfunction	Reflects mucosal permeability and immune activation

**Table 3:** Pathophysiological mechanisms and clinical relevance. Arrows indicate the direction of change: ↑ denotes an increase or upregulation; ↓ denotes a decrease or downregulation.

Mechanism	Molecular Drivers	Histologic Correlate	Clinical Relevance
Epithelial barrier dysfunction [29]	↓ occludin, ↓ claudin, ROS	Metaplasia, loss of cilia	Increased pathogen susceptibility
Immune polarization (Th2) [62]	↑ IL-5, ↑ IL-13	Eosinophilic infiltrate, goblet cell ↑	Responsive to corticosteroids/anti-IL-5 therapy
Fibrosis/remodeling [62,63]	↑ TGF-β, ↑ MMP-9	Lamina propria thickening, loss of glands	May predict recurrence post-surgery
Biofilm persistence [1,2]	TLR stimulation, EPS	Chronic low-grade inflammation	Poor antibiotic response, surgical indication
Hypoxia-induced angiogenesis [35,63]	↑ VEGF, ↑ HIF-1α	Vascular congestion, edema	Surgical outcomes may vary

**Table 4:** Therapeutic strategies based on inflammatory endotypes in chronic nasopharyngeal and otic disorders. Each phenotype is characterized by distinct histological and immunological profiles, guiding specific medical, surgical, or biologic interventions. All references correspond to citations already discussed in the main text. Arrows indicate the direction of change: ↑ denotes an increase or upregulation; ↓ denotes a decrease or downregulation.

Inflammatory Phenotype	Histological Features	Key Markers	Preferred Therapy	Biologic Therapy Options
Eosinophilic	Goblet cell hyperplasia, eosinophil infiltration	IL-5↑, IL-13↑, MBP+, EPO+, Siglec-8^+^ [56,58]	Intranasal corticosteroids, leukotriene antagonists [58]	Anti-IL-5 (benralizumab) [47], anti-IgE (omalizumab) [48], anti-IL-4Rα (dupilumab) [49]
Neutrophilic	Subepithelial neutrophils, biofilm presence	IL-8↑, MMP-9↑, TLR4↑ [34,38]	Topical steroids, antibiotics, surgery for biofilm [12,24]	Experimental (e.g., anti-TNF, anti-IL-17) [59]
Mixed eosinophilic–mast cell	Edema, eosinophils + mast cells	IL-5↑, CCR3^+^, tryptase^+^ [56]	Corticosteroids, antihistamines [58]	Dupilumab [49]
Fibrotic/refractory	Basement membrane thickening, fibrosis, loss of cilia	TGF-β↑, MMP-9↑, FOXJ1↓ [63,64,66]	Surgery (e.g., adenoidectomy, tympanoplasty) [9,76]	Biologic therapy if eosinophilic features persist [49,52]

## Data Availability

All histological images included in this article originate from anonymized archived human tissue samples processed within the Discipline of Histology at the Victor Babeș University of Medicine and Pharmacy Timișoara. The use of these images was approved for academic and scientific purposes by Professor Dr. Flavia Zara, Head of the Discipline of Histology.

## References

[1] Licari A., Magri P., De Silvestri A., Giannetti A., Indolfi C., Mori F., Marseglia G.L., Peroni D. (2023). Epidemiology of Allergic Rhinitis in Children: A Systematic Review and Meta-Analysis. J. Allergy Clin. Immunol. Pract..

[2] Brandtzaeg P. (2011). Potential of Nasopharynx-Associated Lymphoid Tissue for Vaccine Responses in the Airways. Am. J. Respir. Crit. Care Med..

[3] Tang T., Ni X., Song X. (2022). Mucosa-Associated Lymphoid Tissue of Nasopharynx: A Case Report and Literature Review. Radiol. Case Rep..

[4] Alobid I., Armengot-Carceller M., Urraca M.P., Maza-Solano J., Guijarro I.G., Jiménez S.U., Fraile P.S.M., Mullol J. (2024). When the Nose Meets the Lab: Histopathological Analysis in Chronic Rhinosinusitis with Nasal Polyps for Routine Clinical Practice. Curr. Allergy Asthma Rep..

[5] Cassano M., De Corso E., Fiore V., Giancaspro R., Moffa A., Casale M., Trecca E.M.C., Mele D.A., Cassano P., Gelardi M. (2022). Update of Endoscopic Classification System of Adenoid Hypertrophy Based on Clinical Experience on 7621 Children. Acta Otorhinolaryngol. Ital..

[6] Luers J.C., Hüttenbrink K.B. (2016). Surgical Anatomy and Pathology of the Middle Ear. J. Anat..

[7] Cohen D., Raveh D., Peleg U., Nazarian Y., Perez R. (2009). Ventilation and Clearance of the Middle Ear. J. Laryngol. Otol..

[8] Furman D., Campisi J., Verdin E., Carrera-Bastos P., Targ S., Franceschi C., Ferrucci L., Gilroy D.W., Fasano A., Miller G.W. (2019). Chronic Inflammation in the Etiology of Disease across the Life Span. Nat. Med..

[9] Cabral-Pacheco G.A., Garza-Veloz I., Castruita-De la Rosa C., Ramirez-Acuña J.M., Perez-Romero B.A., Guerrero-Rodriguez J.F., Martinez-Avila N., Martinez-Fierro M.L. (2020). The Roles of Matrix Metalloproteinases and Their Inhibitors in Human Diseases. Int. J. Mol. Sci..

[10] Yuan H., Liu J., Gu Y., Ji X., Nan G. (2022). Intermittent Hypoxia Conditioning as a Potential Prevention and Treatment Strategy for Ischemic Stroke: Current Evidence and Future Directions. Front. Neurosci..

[11] Massa H.M., Lim D.J., Kurono Y., Cripps A.W. (2015). Middle Ear and Eustachian Tube Mucosal Immunology. Mucosal Immunology.

[12] Torretta S., Drago L., Marchisio P., Ibba T., Pignataro L. (2019). Role of Biofilms in Children with Chronic Adenoiditis and Middle Ear Disease. J. Clin. Med..

[13] Mendhe S., Badge A., Ugemuge S., Chandi D. (2023). Impact of Biofilms on Chronic Infections and Medical Challenges. Cureus.

[14] Sahni D., Verma P., Bhagat S., Sharma V. (2022). Hearing Assessment in Patients of Allergic Rhinitis: A Study on 200 Subjects. Indian J. Otolaryngol. Head Neck Surg..

[15] Damar M., Dinç A.E., Erdem D., Bişkin S., Eliçora Ş.Ş., Kumbul Y.Ç. (2017). The Role of the Nasal and Paranasal Sinus Pathologies on the Development of Chronic Otitis Media and Its Subtypes: A Computed Tomography Study. Niger. J. Clin. Pract..

[16] Leick M., Azcutia V., Newton G., Luscinskas F.W. (2014). Leukocyte Recruitment in Inflammation: Basic Concepts and New Mechanistic Insights Based on New Models and Microscopic Imaging Technologies. Cell Tissue Res..

[17] Mahapatro M., Erkert L., Becker C. (2021). Cytokine-Mediated Crosstalk between Immune Cells and Epithelial Cells in the Gut. Cells.

[18] Lewis K.L., Del Cid N., Traver D. (2014). Perspectives on Antigen Presenting Cells in Zebrafish. Dev. Comp. Immunol..

[19] Kimura S. (2018). Molecular Insights into the Mechanisms of M-Cell Differentiation and Transcytosis in the Mucosa-Associated Lymphoid Tissues. Anat. Sci. Int..

[20] Casale J., Shumway K.R., Hatcher J.D. (2025). Physiology, Eustachian Tube Function. StatPearls [Internet].

[21] Chauhan G., Tadi P. (2022). Physiology, Postpartum Changes. StatPearls [Internet].

[22] Sundar P.S., Chowdhury C., Kamarthi S. (2021). Evaluation of Human Ear Anatomy and Functionality by Axiomatic Design. Biomimetics.

[23] Mittal R., Lisi C.V., Gerring R., Mittal J., Mathee K., Narasimhan G., Azad R.K., Yao Q., Grati M., Yan D. (2015). Current Concepts in the Pathogenesis and Treatment of Chronic Suppurative Otitis Media. J. Med. Microbiol..

[24] Nistico L., Kreft R., Gieseke A., Coticchia J.M., Burrows A., Khampang P., Liu Y., Kerschner J.E., Post J.C., Lonergan S. (2011). Adenoid Reservoir for Pathogenic Biofilm Bacteria. J. Clin. Microbiol..

[25] Liu Y., Liu S., Meng L., Lin M., Zhai X., Zhang Q., Wang W. (2024). The Function and Mechanism of Human Nasal Mucosa-Derived Mesenchymal Stem Cells in Allergic Rhinitis in Mice. Inflamm. Res..

[26] Mesuraca M., Nisticò C., Lombardo N., Piazzetta G.L., Lobello N., Chiarella E. (2022). Cellular and Biochemical Characterization of Mesenchymal Stem Cells from Killian Nasal Polyp. Int. J. Mol. Sci..

[27] Bhutta M.F., Thornton R.B., Kirkham L.S., Kerschner J.E., Cheeseman M.T. (2017). Understanding the Aetiology and Resolution of Chronic Otitis Media from Animal and Human Studies. Dis. Model. Mech..

[28] Vanneste P., Page C. (2019). Otitis Media with Effusion in Children: Pathophysiology, Diagnosis, and Treatment. A Review. J. Otol..

[29] Brasier A.R. (2025). Interactions between Epithelial Mesenchymal Plasticity, Barrier Dysfunction and Innate Immune Pathways Shape the Genesis of Allergic Airway Disease. Expert Rev. Respir. Med..

[30] Marzoog B.A. (2024). Cytokines and Regulating Epithelial Cell Division. Curr. Drug Targets.

[31] Painter J.T., Clayton N.P., Herbert R.A. (2009). Useful Immunohistochemical Markers of Tumor Differentiation. Toxicol. Pathol..

[32] Kuo W.T., Odenwald M.A., Turner J.R., Zuo L. (2022). Tight Junction Proteins Occludin and ZO-1 as Regulators of Epithelial Proliferation and Survival. Ann. N. Y. Acad. Sci..

[33] Fujieda S., Imoto Y., Kato Y., Ninomiya T., Tokunaga T., Tsutsumiuchi T., Yoshida K., Kidoguchi M., Takabayashi T. (2019). Eosinophilic Chronic Rhinosinusitis. Allergol. Int..

[34] Alharbi T.A.F., Rababa M., Alsuwayl H., Alsubail A., Alenizi W.S. (2025). Diagnostic Challenges and Patient Safety: The Critical Role of Accuracy—A Systematic Review. J. Multidiscip. Healthc..

[35] Malkov M.I., Lee C.T., Taylor C.T. (2021). Regulation of the Hypoxia-Inducible Factor (HIF) by Pro-Inflammatory Cytokines. Cells.

[36] Saha S., Müller D., Clark A.G. (2023). Mechanosensory Feedback Loops during Chronic Inflammation. Front. Cell Dev. Biol..

[37] Deshmane S.L., Kremlev S., Amini S., Sawaya B.E. (2009). Monocyte Chemoattractant Protein-1 (MCP-1): An Overview. J. Interf. Cytokine Res..

[38] Patel G.B., Kern R.C., Bernstein J.A., Hae-Sim P., Peters A.T. (2020). Current and Future Treatments of Rhinitis and Sinusitis. J. Allergy Clin. Immunol. Pract..

[39] Ross K.F., Herzberg M.C. (2016). Autonomous Immunity in Mucosal Epithelial Cells: Fortifying the Barrier against Infection. Microbes Infect..

[40] Rather M.A., Gupta K., Mandal M. (2021). Microbial Biofilm: Formation, Architecture, Antibiotic Resistance, and Control Strategies. Braz. J. Microbiol..

[41] Pat Y., Yazici D., D’Avino P., Li M., Ardicli S., Ardicli O., Mitamura Y., Akdis M., Dhir R., Nadeau K. (2024). Recent Advances in the Epithelial Barrier Theory. Int. Immunol..

[42] Lee H.S., Kim W.J. (2022). The Role of Matrix Metalloproteinase in Inflammation with a Focus on Infectious Diseases. Int. J. Mol. Sci..

[43] Budi E.H., Schaub J.R., Decaris M., Turner S., Derynck R. (2021). TGF-β as a Driver of Fibrosis: Physiological Roles and Therapeutic Opportunities. J. Pathol..

[44] Cross M.J., Claesson-Welsh L. (2001). FGF and VEGF Function in Angiogenesis: Signalling Pathways, Biological Responses and Therapeutic Inhibition. Trends Pharmacol. Sci..

[45] Klibaner-Schiff E., Simonin E.M., Akdis C.A., Cheong A., Johnson M.M., Karagas M.R., Kirsh S., Kline O., Mazumdar M., Oken E. (2024). Environmental Exposures Influence Multigenerational Epigenetic Transmission. Clin. Epigenetics.

[46] Yu K., Tenaglia V., Chua E.G., Haines R., Bahal G., Nicol M.P., Bahal R.K. (2025). Interactions between Bacteria in the Human Nasopharynx: A Scoping Review. Lancet Microbe.

[47] Chiner E., Murcia M., Boira I., Bernabeu M.Á., Esteban V., Martínez-Moragón E. (2024). Real-Life Clinical Outcomes of Benralizumab Treatment in Patients with Uncontrolled Severe Asthma and Coexisting Chronic Rhinosinusitis with Nasal Polyposis. J. Clin. Med..

[48] Gevaert P., Omachi T.A., Corren J., Mullol J., Han J., Lee S.E., Kaufman D., Ligueros-Saylan M., Howard M., Zhu R. (2020). Efficacy and Safety of Omalizumab in Nasal Polyposis: Two Randomized Phase 3 Trials. J. Allergy Clin. Immunol..

[49] Bachert C., Han J.K., Desrosiers M., Hellings P.W., Amin N., Lee S.E., Mullol J., Greos L.S., Bosso J.V., Laidlaw T.M. (2019). Efficacy and Safety of Dupilumab in Patients with Severe Chronic Rhinosinusitis with Nasal Polyps (LIBERTY NP SINUS-24 and LIBERTY NP SINUS-52): Results from Two Multicentre, Randomised, Double-Blind, Placebo-Controlled, Parallel-Group Phase 3 Trials. Lancet.

[50] Subramanian D., Cruz C.V., Garcia-Bournissen F. (2022). Systematic Review of Early Phase Pediatric Clinical Pharmacology Trials. J. Pediatr. Pharmacol. Ther..

[51] Wedner H.J., Fujisawa T., Guilbert T.W., Ikeda M., Mehta V., Tam J.S., Lukka P.B., Asimus S., Durżyński T., Johnston J. (2024). Benralizumab in Children with Severe Eosinophilic Asthma: Pharmacokinetics and Long-Term Safety (TATE Study). Pediatr. Allergy Immunol..

[52] Hillson K., Saglani S., Bush A. (2024). The New Biologic Drugs: Which Children with Asthma Should Get What?. Pediatr. Pulmonol..

[53] Kim S.W., Roh J., Park C.S. (2016). Immunohistochemistry for Pathologists: Protocols, Pitfalls, and Tips. J. Pathol. Transl. Med..

[54] Pahwa R., Goyal A., Jialal I. (2025). Chronic Inflammation. StatPearls [Internet].

[55] Pavlasova G., Mraz M. (2020). The Regulation and Function of CD20: An “Enigma” of B-Cell Biology and Targeted Therapy. Haematologica.

[56] Kuang F.L. (2020). Approach to Patients with Eosinophilia. Med. Clin. N. Am..

[57] Li A., Yang D.H. (2020). Application of Immunohistochemistry in Basic and Clinical Studies. Methods in Molecular Biology.

[58] Eyerich K., Dimartino V., Cavani A. (2017). IL-17 and IL-22 in Immunity: Driving Protection and Pathology. Eur. J. Immunol..

[59] Alam M.S., Otsuka S., Wong N., Abbasi A., Gaida M.M., Fan Y., Meerzaman D., Ashwell J.D. (2021). TNF Plays a Crucial Role in Inflammation by Signaling via T Cell TNFR2. Proc. Natl. Acad. Sci. USA.

[60] Hamed M.A., Nakata S., Sayed R.H., Ueda H., Badawy B.S., Nishimura Y., Kojima T., Iwata N., Ahmed A.R., Dahy K. (2016). Pathogenesis and Bone Resorption in Acquired Cholesteatoma: Current Knowledge and Future Prospectives. Clin. Exp. Otorhinolaryngol..

[61] Ott L.W., Resing K.A., Sizemore A.W., Heyen J.W., Cocklin R.R., Pedrick N.M., Woods H.C., Chen J.Y., Goebl M.G., Witzmann F.A. (2007). Tumor Necrosis Factor-Alpha- and Interleukin-1-Induced Cellular Responses: Coupling Proteomic and Genomic Information. J. Proteome Res..

[62] Lu P., Takai K., Weaver V.M., Werb Z. (2011). Extracellular Matrix Degradation and Remodeling in Development and Disease. Cold Spring Harb. Perspect. Biol..

[63] Ahmad Z., Krüger K., Lautermann J., Lippert B., Tenenbaum T., Tigges M., Tisch M. (2023). Adenoid Hypertrophy—Diagnosis and Treatment: The New S2k Guideline. HNO.

[64] Wang H., Bai J., Zhang J., Yang W., Zuo K., Li H. (2013). IL-6 Promotes the Expression of Vascular Endothelial Growth Factor through the p38 Signalling Pathway in Hypertrophied Adenoids in Children. Int. J. Pediatr. Otorhinolaryngol..

[65] Kayama H., Takeda K. (2020). Manipulation of Epithelial Integrity and Mucosal Immunity by Host and Microbiota-Derived Metabolites. Eur. J. Immunol..

[66] Coopman P., Djiane A. (2016). Adherens Junction and E-Cadherin Complex Regulation by Epithelial Polarity. Cell Mol. Life Sci..

[67] Doran E., Cai F., Holweg C.T.J., Wong K., Brumm J., Arron J.R. (2017). Interleukin-13 in Asthma and Other Eosinophilic Disorders. Front. Med..

[68] Pant H., Hughes A., Schembri M., Miljkovic D., Krumbiegel D. (2014). CD4(+) and CD8(+) Regulatory T Cells in Chronic Rhinosinusitis Mucosa. Am. J. Rhinol. Allergy.

[69] Ryu G., Bae J.S., Yoo S.H., Kim D.K., Han D.H., Mo J.H., Cho S.H., Jin H.R. (2025). Elevated IL-17A-Secreting Regulatory T Cells in Sinonasal Tissues of Chronic Rhinosinusitis with Nasal Polyps. Inflammation.

[70] Poposki J.A., Klingler A.I., Stevens W.W., Suh L.A., Tan B.K., Peters A.T., Abdala-Valencia H., Grammer L.C., Welch K.C., Smith S.S. (2022). Elevation of Activated Neutrophils in Chronic Rhinosinusitis with Nasal Polyps. J. Allergy Clin. Immunol..

[71] Caruso C., Giancaspro R., Guida G., Macchi A., Landi M., Heffler E., Gelardi M. (2022). Nasal Cytology: A Easy Diagnostic Tool in Precision Medicine for Inflammation in Epithelial Barrier Damage in the Nose. A Perspective Mini Review. Front. Allergy.

[72] Danisman Z., Linxweiler M., Kühn J.P., Linxweiler B., Solomayer E.F., Wagner M., Wagenpfeil G., Schick B., Berndt S. (2023). Differential Nasal Swab Cytology Represents a Valuable Tool for Therapy Monitoring but Not Prediction of Therapy Response in Chronic Rhinosinusitis with Nasal Polyps Treated with Dupilumab. Front. Immunol..

[73] Artono A., Purnami N., Handoko E., Widodo A.D.W., Juniastuti J. (2025). *Pseudomonas aeruginosa* in Chronic Suppurative Otitis Media. Infect. Chemother..

[74] Endo L.H., Vassallo J., Sakano E., Brousset P. (2002). Detection of Epstein-Barr Virus and Subsets of Lymphoid Cells in Adenoid Tissue of Children under 2 Years of Age. Int. J. Pediatr. Otorhinolaryngol..

[75] Hsueh C.Y., Yang C.F., Gau J.P., Kuan E.C., Ho C.Y., Chiou T.J., Hsiao L.T., Lin T.A., Lan M.Y. (2019). Nasopharyngeal Lymphoma: A 22-Year Review of 35 Cases. J. Clin. Med..

[76] Abdel Aziz A.A.R., Youssef A.M., Mostafa M.M., Talaat M., Abdelzaher K.M., Sadeq A.A. (2022). Cartilage Tympanoplasty in the Treatment of Adhesive Otitis Media with and without Eustachian Tube Balloon Dilatation. J. Otol..

[77] Zou X.L., Yang H.L., Ding W.W., Li H.K., Zhou Y.Q., Zhang T.T. (2023). Down-Expression of Foxj1 on Airway Epithelium with Impaired Cilia Architecture in Non-Cystic Fibrosis Bronchiectasis Implies Disease Severity. Clin. Respir. J..

